# Resveratrol: A Potential Hippocampal Plasticity Enhancer

**DOI:** 10.1155/2016/9651236

**Published:** 2016-05-25

**Authors:** Gisele Pereira Dias, Graham Cocks, Mário Cesar do Nascimento Bevilaqua, Antonio Egidio Nardi, Sandrine Thuret

**Affiliations:** ^1^Translational Neurobiology Unit, Laboratory of Panic & Respiration/Institute of Psychiatry, Universidade Federal do Rio de Janeiro, Avenida Venceslau Brás, 71 Fundos, 22290-140 Praia Vermelha, RJ, Brazil; ^2^Department of Basic and Clinical Neuroscience/Institute of Psychiatry, Psychology and Neuroscience, King's College London, London SE5 9NU, UK; ^3^The Maurice Wohl Clinical Neuroscience Institute, 125 Coldharbour Lane, London SE5 9NU, UK; ^4^Health and Environment School, Universidade Castelo Branco, Avenida de Santa Cruz 1631, 21710-255 Realengo, RJ, Brazil

## Abstract

The search for molecules capable of restoring altered hippocampal plasticity in psychiatric and neurological conditions is one of the most important tasks of modern neuroscience. It is well established that neural plasticity, such as the ability of the postnatal hippocampus to continuously generate newly functional neurons throughout life, a process called adult hippocampal neurogenesis (AHN), can be modulated not only by pharmacological agents, physical exercise, and environmental enrichment, but also by “nutraceutical” agents. In this review we focus on resveratrol, a phenol and phytoalexin found in the skin of grapes and red berries, as well as in nuts. Resveratrol has been reported to have antioxidant and antitumor properties, but its effects as a neural plasticity inducer are still debated. The current review examines recent evidence implicating resveratrol in regulating hippocampal neural plasticity and in mitigating the effects of various disorders and diseases on this important brain structure. Overall, findings show that resveratrol can improve cognition and mood and enhance hippocampal plasticity and AHN; however, some studies report opposite effects, with resveratrol inhibiting aspects of AHN. Therefore, further investigation is needed to resolve these controversies before resveratrol can be established as a safe coadjuvant in preventing and treating neuropsychiatric conditions.

## 1. Introduction

**Figure 1:**
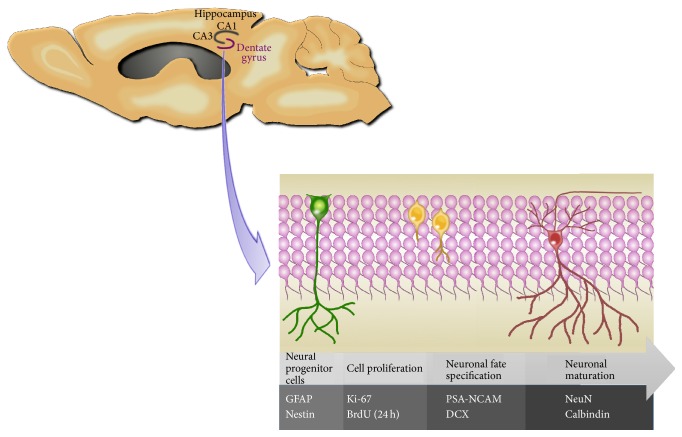
Adult hippocampal neurogenesis markers. AHN is a highly regulated process that occurs in stages. In this sense, the pool of NPCs expressing markers such as GFAP and nestin is under constant self-renewal through regulated proliferative activity. Proliferation can be assessed by the number of cells expressing the cell division markers Ki-67 and BrdU (in this case, when the tissue is fixed 24 h following the last injection of BrdU). Next, the cells undergo the stage of fate specification when they express PSA-NCAM and DCX, if given the specific molecular signaling to commit into a neuronal lineage. After this stage of fate commitment, the newborn neurons undergo maturation and express the neuronal markers NeuN and calbindin. AHN = adult hippocampal neurogenesis; BrdU = bromodeoxyuridine; DCX = doublecortin; GFAP = glial fibrillary acidic protein; NeuN = neuronal nuclei; NPCs = neural progenitor cells; and PSA-NCAM = polysialylated neuronal cell adhesion molecule.

Neural plasticity refers to the ability of the nervous system to adaptively respond to changes in the environment sensed by the organism and extends to stimuli such as an enriched environment (EE), increased physical activity, and changes in pharmaceutical and nutritional intake (reviewed in [1–4]). Among the most remarkable forms of neural plasticity is the capacity of the adult hippocampus to continuously generate functional neurons throughout life, a process known as adult hippocampal neurogenesis (AHN). Neurogenesis in the adult hippocampus is only possible due to the presence of a pool of neural progenitor cells (NPCs) under constant self-renewal in the subgranular zone (SGZ) of the dentate gyrus (DG) of the hippocampus (reviewed in [5]). AHN is a highly regulated process, encompassing stages of cell proliferation, neuronal differentiation, maturation, survival, and functional integration into preexisting circuits (Figure 1). In each of these stages, cells express specific markers, allowing researchers to characterize AHN-related endophenotypes of a certain pathological condition or to identify the specific stages where a proposed intervention exerts its effects.

The search for molecules and lifestyle changes that are able to restore AHN in incapacitating and highly prevalent conditions, such as Alzheimer's disease (AD) [6], depression [7–9], anxiety [10], stroke [11], diabetes [12], and chemotherapy-induced cognitive impairment (reviewed in [13]), is one of the most challenging and relevant goals of modern neuroscience. Here we examine the role of nutrition as a potential enhancer of AHN. In this respect, a number of studies have shown that total calorie intake, as well as meal frequency, texture, and content are able to modulate AHN (reviewed in [14]). Particularly in the context of food content, a growing literature point to polyphenols—compounds widely found in certain fruits, spices, and tea leaves—as capable of inducing important antioxidant responses in the brain [15], as well as protecting or enhancing AHN levels [16–18] (reviewed in [19]).

Within the context of polyphenols, we focus here on resveratrol (3,5,4′-trihydroxy-*trans*-stilbene; RSV), a phenol and phytoalexin found in the skin of grapes and red berries, as well as in several types of nut. RSV is thought to exert part of its actions through the activation of the histone deacetylase enzyme sirtuin 1 (silent mating type information regulation 2 homolog; SIRT1) [20–22]. In addition, its neuroprotective effects appear to be mediated by an increase in the activation of AMP-activated kinase (AMPK), leading to neurite outgrowth and stimulation of mitochondrial biogenesis [23]. RSV's antioxidant [24] (reviewed in [25]), anti-inflammatory [26], and antitumor [27–29] activities are well documented. RSV is also known for its ability to promote increased lifespan [30, 31], resembling the effects exerted by caloric restriction (CR). However, with regard to AHN enhancement, studies show opposing and contradictory results, and therefore it is still intensely debated whether RSV can be considered a proplasticity inducer in the context of AHN. This systematic review aims to describe and discuss the most recent findings on the effects of RSV on AHN, so that a clearer picture of the circumstances in which this polyphenol may exert either pro- or antiplasticity effects at the level of AHN can be delineated.

## 2. Methods

A bibliographical search was carried out in the databases Medline/PubMed and Web of Science/ISI in order to collect studies about RSV, hippocampal neurogenesis, and hippocampal plasticity. The keywords used were “resveratrol AND hippocampal neurogenesis” and “resveratrol AND hippocampal plasticity”. The search results are displayed in Table 1. Only original papers published in English from 2000 to 2015 and directly assessing hippocampal neurogenesis or other hippocampal plasticity markers accompanied by treatment with RSV were selected. Eleven articles following these criteria were identified, both in vivo and in vitro, to compose Table 2. Seven papers that did not appear in the original search-engine results were further identified manually to comply with the inclusion criteria and were added to the selected literature.

## 3. Results and Discussion

Results are discussed in accordance with the disorder or condition investigated: stress, ethanol-induced toxicity, chronic fatigue, stroke, diabetes, aging, AD, and the healthy brain. Main findings are shown in Table 2 and discussed in the following sections.

### 3.1. RSV and Stress

Stress has been widely proposed as an important risk factor for depression. A range of evidence supports this idea, with one of the most widely accepted animal models for depression being generated as a result of chronic submission to several different and unpredictable stressors (unpredictable chronic mild stress [UCMS]), proposed by Willner et al. in 1987 [32]. Rodents submitted to the UCMS paradigm display depressive-related phenotypes, such as reduced consumption of sucrose [33], increased immobility time in the forced swimming test (FST) [33, 34], impairment of coat state [35] and of hippocampal-dependent learning [36]. Intriguingly, the behavioral changes triggered by exposition to stressful events are often followed by a number of hippocampal alterations, including reduced levels of AHN [36, 37] and brain-derived neurotrophic factor (BDNF) [37]. As demonstrated in several studies, at certain doses, RSV can exert antidepressant effects and restore the hippocampal plasticity markers which are found to be decreased in stress-induced models of depression. For instance, intraperitoneal injections of RSV (mainly at 80 mg/kg; see Table 2 for details) had similar effects to those of antidepressant desipramine, both in normalizing behavior and serum corticosterone (CORT) levels in rats exposed to UCMS for 5 weeks [38]. The study also revealed that RSV could restore the levels of BDNF, phosphorylated extracellular signal-regulated kinase (pERK), a protein in the ERK pathway, involved in the differentiation, survival, and other aspects of neuronal plasticity [39], and phosphorylated cAMP response element-binding protein (pCREB), involved in enhancing the transcription of the BDNF gene [38]. Similarly, the same regimen of RSV was shown to prevent the cognitive deficits caused by the UCMS in the Morris water maze (MWM) and in the novel object recognition task, also accompanied by restoration of BDNF, pERK, and pCREB levels [40]. In another report, RSV at a dose of just 20 mg/kg was found to prevent the UCMS-induced cognitive impairments in the MWM, and also in the passive-avoidance test, a test was designed to assess emotional memory [41]. Once again, the neuroprotective effects of RSV against the deficits induced by the UCMS paradigm were proposed to be mediated by restored levels of hippocampal BDNF (as shown in CA1 (cornus ammonis 1) and CA3 regions). In addition, the reduction in c-Fos protein expression following UCMS was prevented by RSV, suggesting that the effects of this polyphenol can include changes in target gene expression. Furthermore, the study reports anti-inflammatory effects of RSV, in that it normalized the UCMS-induced higher circulating levels of tumor necrosis factor-*α* (TNF-*α*) and interleukin-1*β* (IL-1*β*). Considering the detrimental effects of both TNF-*α* [42] and IL-1*β* [43] (reviewed in [44]) over parameters of hippocampal neurogenesis, it is plausible to reason that, by decreasing the levels of these proinflammatory cytokines, RSV could contribute to preserving neurogenesis and, therefore, protecting cognitive function. Also in the context of inflammation, Bellaver et al. showed that RSV could prevent the decrease in antioxidant defenses and the increase in inflammatory responses (as measured by the levels of TNF-*α* and IL-1*β*) in hippocampal astrocyte cultures of adult and aged Wistar rats [45]. Although the study did not investigate parameters of hippocampal neurogenesis, it is well known that astrocytes play an important role in the regulation of a number of neural plasticity events, including neurogenesis [46]. Therefore, a potential protection of AHN by RSV through astrocytic regulation should not be discarded.

Besides the UCMS paradigm, another way to induce depressive-like behaviors in rodents is by repeated administration of CORT. A recent study used this model and obtained a depressive-like phenotype in mice after 21 days of CORT subcutaneous injection (40 mg/kg) [47]. Interestingly, orally administered RSV (80 mg/kg) 30 min prior to the 21 CORT injections was able to significantly ameliorate all the behavioral parameters analyzed, including RSV increased sucrose consumption and decreased immobility time both in the FST and in the tail suspension test (TST), to levels comparable with those found in CORT + fluoxetine-treated mice [47]. Moreover, hippocampal BDNF levels were found to be increased in CORT + RSV- and CORT + fluoxetine-treated animals. Although these findings are consistent and encouraging, other hippocampal plasticity markers (such as those related to AHN) have not been investigated, indicating the need for further studies.

In the context of stress, a study investigated the potential neuroprotective effects of RSV (10 mg/kg body weight) orally administered throughout pregnancy in the offspring of female rats subjected to restraint stress (3 times a day, for 45 minutes) in either the early (gestation day 1 to 10) or late (gestation day 11 till delivery) gestational periods [48]. The study demonstrated that prenatal administration of RSV was neuroprotective for the offspring at postnatal day 40 (PND40) from the deleterious effects of prenatal stress on anxiety (as measured in the open field test) and on cognitive function (as assessed in the MWM). The study, however, did not investigate hippocampal plasticity parameters. Considering the involvement of the hippocampus in both anxiety and cognitive regulation, it is clearly of interest to further investigate hippocampal-related plasticity markers in a similar experimental design, and a subsequent study by Madhyastha et al. [49] attempted to address this issue. Using the same prenatal stress and RSV regimen, the authors found that this polyphenol was able to improve the number of DCX^+^ (doublecortin) neurons in the DG, as well as hippocampal BDNF expression, of PND40 pups whose dams have been exposed to prenatal stress.

Interestingly, it is not only in stress-induced models of dysfunctional phenotypes that RSV has been shown to exert positive effects. In Wistar-Kyoto rats, an inbred strain of rodents that displays depressive-like behavior even in the absence of aversive stimuli, RSV intraperitoneally administered acutely (40 mg/kg) and for 7 days (10 mg/kg and 40 mg/kg) could reduce immobility time in the FST [50]. The chronic regimen, at both doses, could also increase sucrose intake. Interestingly, at one-week posttreatment, behavioral effects could be no longer observed, suggesting that sustained consumption of RSV might be necessary for antidepressant effects. With regard to plasticity markers, following 7 days of RSV treatment (10 mg/kg and 40 mg/kg), BDNF has been found to be increased in the hippocampus, an effect not observed in other brain areas involved in the neurobiology of depression, such as the frontal cortex.

### 3.2. RSV and Ethanol-Induced Toxicity

Ethanol (EtOH) exposure in utero is well recognized as an important risk factor in abnormal brain development and function. Indeed, a number of adverse outcomes at the cognitive, physical, and behavioral levels have been described as a result of prenatal EtOH exposure, giving rise to a continuum of conditions known as Fetal Alcohol Spectrum Disorders (FASD) [51]. The detrimental effects of alcohol to the brain and hippocampal plasticity, however, are not restricted to exposure during the prenatal period. In the rat DG, for instance, EtOH administered at the end of the first postnatal week (PND7) was capable of significantly reducing the pool of neural stem cells (NSCs) and NPCs [52], a finding that may have subsequent consequences for AHN.

In order to investigate the putative role of RSV in protecting the neonatal hippocampus against the deleterious effects of EtOH, a recent report pretreated C57/BL6 mice at age PND6 with RSV (20 mg/kg), subsequently exposing them to 20% EtOH (total of 5 g/kg) at PND7 [53]. The study investigated a number of hippocampal neurogenesis markers, as well as other aspects of neural plasticity. For instance, using bromodeoxyuridine (BrdU) labeling to assess cell proliferation at PND8, it was found that RSV could protect the neonatal DG, reversing the EtOH-induced reduction in cell proliferation. Neuroprotective effects of RSV were also observed with regard to attenuation of the decreased pool of hippocampal neural precursor cells, as shown by the number of Sox2^+^, Sox2/glial fibrillary acidic protein^+^ (GFAP), and brain lipid-binding protein (BLBP)/nestin+ cells in the DG. RSV was also found to reverse the antineurogenic effects of EtOH at PND14, as measured by the number of cells expressing both BrdU and DCX. In addition, pretreatment with this polyphenol could promote the reversal of the reduced spine density of granule neurons in mice also exposed to EtOH. In fact, not only the density of spines was augmented as a result of RSV treatment prior to EtOH exposure, but also the proportion of more mature, mushroom shaped spines was found to be higher in RSV-treated groups. Hippocampal levels of proteins involved in the proliferation, maintenance, and fate determination of NPCs, such as pERK [54], Hes 1 (hairy and enhancer of split-1) [55], and Sirt1 [56], were also verified to be increased in the RSV + EtOH group. Interestingly, the findings of RSV treatment in vivo on cell proliferation were also consistent with those of in vitro assays. Using C17.2 NPCs cells, the same study reported that pretreatment with RSV could attenuate the detrimental effects of EtOH on the number of Ki-67^+^ cells, as well as reducing apoptosis and preventing the cell cycle arrest mediated by EtOH exposure [53].

### 3.3. RSV and Chronic Fatigue

Another condition whose pathophysiology has been found to include hippocampal abnormalities is chronic fatigue syndrome (CFS). For instance, reduced levels of* N*-acetylaspartate, a marker of neuronal metabolism, were found in the right hippocampus of CFS patients [57]. Other lines of evidence linking the hippocampus and CFS come from studies reporting a particular reduction in serotonin 5-HT1A receptor binding potential in this brain structure in these individuals [58], as well as a significant increase in blood oxygen level dependent (BOLD) activity in brain regions including the hippocampus of CFS subjects during a fatiguing cognitive task [59].

In the context of RSV, Moriya et al. [60] found that daily doses of orally administered RSV (40 mg/kg) for 4 weeks could rescue the decreased daily activity of an animal model of CSF. At the hippocampal level, this behavioral effect was accompanied by an increase in cell proliferation in the DG, as measured by BrdU labeling, and a decrease in the levels of apoptosis, as measured by terminal deoxynucleotidyl transferase dUTP nick end labeling assay (TUNEL) in the DG, as also suggested by the reduction in the levels of acetylated p53 in the hippocampus.

### 3.4. RSV and Stroke

In pathological conditions such as stroke, quiescent NSCs can become active, a phenomenon that is being actively investigated in the field of neural repair and regeneration. Also, in the context of stroke, RSV has emerged as a potential plasticity inducer, with evidence pointing to an antiapoptotic action in hippocampal neurons after focal cerebral ischemia in rats [61], and by attenuation of the cerebral ischemic injury through upregulation of transcription factor nuclear factor erythroid 2-related factor 2 (Nrf-2) and enzyme heme oxygenase 1 (HO-1) [62], implicated in oxidative stress responses [63].

With regard to hippocampal neurogenesis, in an in vitro model of stroke using oxygen-glucose deprivation/reoxygenation (OGD/R), pretreatment with RSV was able to increase NSCs survival and proliferation [64]. Furthermore, RSV administered prior to the insult was associated with upregulation of protein patched homolog 1 (Patched-1), Smoothened (Smo) and Gli-1 proteins, and mRNA, indicating that RSV effects in this condition were mediated by sonic hedgehog signaling. These findings, however promising, derived from cultured cerebral cortices of rats and could not be directly extrapolated to the context of AHN. Another recent study, nevertheless, analyzed the rat hippocampus following global cerebral ischemia and previous treatment with RSV [65]. The authors found that RSV (at 1 and 10 mg/kg) could protect CA1 neurons from the ischemic insult at both 7 and 85 days after surgery. Protein platelet endothelial cell adhesion molecule-1 (PECAM-1) (CD31), a selective marker of angiogenesis, has also been found at higher density in hippocampal area CA1 (1 mg/kg, 7 days after ischemia; 1 and 10 mg/kg, at both 7 and 85 days after ischemia), CA3 (1 and 10 mg/kg, 7 days after ischemia), and DG (1 and 10 mg/kg, 85 days after ischemia). This latter finding deserves special attention, considering the association between local angiogenesis and normal levels of AHN [66]. Specifically, with regard to AHN, Girbovan et al. [65] found that RSV treatment prior to the global cerebral ischemia was associated with reduced number of DCX-PSA-NCAM colabeling cells at both doses and intervals studied. According to the authors, one possible explanation for this intriguing finding is that RSV can decrease microglia and astrocyte activation 7 days after the ischemic insult [67], which can therefore inhibit glial released trophic factor-induced neurogenesis [68]. However, despite the reduced AHN found, increased swimming time in the target quadrant during the probe trial of the MWM was found in RSV-treated ischemic rats. Further studies are therefore warranted so that a better understanding of the effects of RSV as an inducer or inhibitor of plasticity at the AHN level in the context of stroke can be achieved.

Some of the most interesting findings relating RSV to hippocampal plasticity within the context of ischemia come from measures of poststroke depression. A recent study showed that oral administration of RSV (20 and 40 mg/kg) was able to significantly reduce the infarction volume of the brain 22 h following middle cerebral artery occlusion (MCAO) and exerted antidepressant effects 13 days after insult [69]. These antidepressant effects included increased sucrose preference and decreased immobility time in the FST to levels comparable to those elicited by antidepressant imipramine. At the hippocampal level, the authors found that the aforementioned doses of RSV were able to decrease the levels of corticotropin-releasing factor (CRF) as well as to increase the expression of glucocorticoid receptors (GR), both measures indicating normalized activation of the hypothalamic-pituitary-adrenal (HPA) axis. Moreover, hippocampal levels of BDNF protein were found to be increased in MCAO rats treated with RSV. It would have been interesting if the study had also included the analysis of neurogenic markers; considering that AHN can be reduced when the HPA axis is activated [70] and, conversely, it is normally found to be increased upon higher levels of hippocampal BDNF (reviewed in [71]), it is plausible to hypothesize that RSV could augment AHN in MCAO rats. Empirical evidence to assert this hypothesis is, nevertheless, needed.

### 3.5. RSV and Diabetes

Due to its association with cognitive deficits (reviewed in [72]), the link between diabetes and hippocampal changes has been receiving growing attention in the past few years. In clinical studies, it has been shown that patients with type-2 diabetes (T2D) exhibit cognitive deficits which are associated with changes in left hippocampal metabolism [73]. Animal models of diabetes also display a number of hippocampal changes such as synaptic integrity loss, as measured by a decrease in the levels of postsynaptic density protein 95 (PSD-95) and synaptosomal-associated protein 25 (SNAP 25) in the hippocampus of T2D mice [74].

With regard to RSV, this polyphenol has been found to attenuate a number of diabetes-induced neurodegenerative markers. For instance, Jing et al. used the streptozotocin-induced diabetes model and found that the number of degenerative neurons in CA3 was increased, as well as astrocytic activation in CA1 and CA3 and hippocampal expression of TNF-*α*, IL-6, pERK1/2, and phospho-p38, among others [75]. All these parameters were significantly restored following oral administration of RSV (0.75 mg/kg) 3 times per day for 4 weeks. Although the study did not investigate AHN markers, it is plausible to reason that neurogenesis is likely to be disrupted in the model, especially considering the increased levels of hippocampal proinflammatory markers found. Supporting evidence came from a study by Thomas et al. [76] where 6 weeks of RSV supplementation (50 mg/kg) in mice resulted in normalization of expression of genes implicated with hippocampal neurogenesis and synaptic plasticity (such as Hdac4, Hat1, Wnt7a, and ApoE), which had been previously found to be altered as a consequence of the diabetic state. Whether a similar RSV regimen could indeed ameliorate the possible diabetes-mediated disruption in AHN is a question yet to be answered, especially considering that other supplementation regimens in rats (20 mg/kg for 21 days) were not able to restore the lower cell proliferation levels found in the hippocampus of diabetic rats [77]. Also reinforcing the idea that RSV can potentially protect cognitive function in the context of impaired glucose metabolism, Palomera-Avalos et al. used the Senescence-accelerated prone mouse model (SAMP8, a model of glucose hypometabolism characteristic of aging and AD) and showed that RSV added to a high-fat diet (HF) for 15 weeks could prevent the behavioral deficits observed in SAMP8 mice subjected to HF [78]. In particular, it was shown that RSV could prevent the deleterious effects of HF-induced metabolic stress on the novel object recognition test (NORT) and the probe trial of the MWM. The study also showed that RSV could restore mitochondrial function and reduce oxidative stress and parameters of AD, such as Tau hyperphosphorylation. Moreover, the authors showed that in HF-fed SAMP8 mice RSV promoted action of the Wnt pathway, which is known to be important for AHN [79]. Further studies on the effects of RSV in the context of aging and AD will be discussed next.

### 3.6. RSV, Aging, and AD

AD is one of the most incapacitating of neuropsychiatric conditions, posing important emotional, social, and financial burdens on patients, carers, and society in general. The demographic shift to a higher proportion of older people, particularly in the developed world, places a special urgency on unravelling etiological aspects of the disease and effective ways to intervene and interrupt its progression. Although the search for effective pharmaceuticals is the basis of this endeavor, nutritional supplementation also arises as a potential coadjuvant for both prevention and treatment of AD (reviewed in [80, 81]).

In order to investigate the potential neuroprotective effects of RSV in the context of AD, a recent in vitro study pretreated rat hippocampal neuronal cells with RSV (75 *μ*M) for 2 h followed by 24 h of incubation with A*β* (25 *μ*M) [82]. The findings are encouraging, in that RSV was able to attenuate lipid peroxidation in A*β*-treated cells and restore a number of other oxidative damage markers, improving the levels of ascorbic acid, glutathione reductase, superoxide dismutase, among others. Of special interest here, pretreatment with RSV was also able to improve the hippocampal levels of the synaptic proteins PSD-95, synaptophysin, and activity-regulated cytoskeleton-associated protein (Arc).

Promoting healthy aging could be one of the ways to prevent or delay the onset of AD. Within the context of healthy aging, a recent report by Kodali et al. [83] showed that a 4-week treatment with RSV (40 mg/kg) at the age of 21 months brought a series of beneficial effects at 25 months of age, both at the behavioral and hippocampal levels, in comparison with same-age vehicle-treated rats. Among the behavioral effects, RSV-treated rodents displayed decreased latency to reach the hidden platform of the MWM, as well as having improved memory in the probe trial. Antidepressant effects were also identified, as shown by the decreased floating time in the FST. These cognition- and mood-related behavioral outcomes were accompanied by improvements in hippocampal plasticity markers, such as increased number of BrdU^+^ and DCX^+^ cells in the SGZ-granule cell layer (GCL), increased net neurogenesis (defined as the number of BrdU^+^ cells with the percentage of newborn cells also expressing neuronal nuclei protein, NeuN), and enhanced microvasculature, as shown by rat endothelial cell antigen-1 (RECA-1) immunostaining in CA1 and entire hippocampus. Furthermore, the authors demonstrated that the hippocampi of RSV-treated aged rats displayed reduced hypertrophy of astrocytes and reduced microglia activation, suggesting that this polyphenol is able to diminish the chronic low-level inflammation found in the aging rat brain. Although the focus of the study was not on dementia, considering the interplay between neuroinflammation and AD (reviewed in [84]), the findings by Kodali et al. [83] suggest that further testing of RSV in animal models of AD could be valuable.

### 3.7. RSV and the Healthy Brain

As discussed by Girbovan et al. [65], not many studies have investigated the effects of RSV consumptions under nonpathological conditions. This is particularly important, considering the need to better understand under which circumstances this polyphenol could exert beneficial effects to brain health and plasticity.

In this regard, Torres et al. [85] have demonstrated that dietary supplementation with RSV leads to increased cell proliferation in the DG, as determined by the number of Ki-67^+^ cells, as well as to an increase in the expression of presenilin 1, a regulator of AHN [86] and also involved in AD pathogenesis [87, 88] (reviewed in [89]). Also of interest to hippocampal plasticity and AHN, the authors found that dietary supplementation with RSV was associated with increased expression of the transcriptional repressor Hes 1, involved in stem cell maintenance through the Notch homolog 1 (NOTCH1) signaling pathway. At the behavioral level, an 18-month treatment with RSV (200 mg/kg/day) was capable of improving working memory in the spontaneous alternation task in nonhuman primates to levels comparable with those of CR-treated animals [90]. In addition, supplementation with RSV—but not the CR regimen—led to increased spatial memory in the circular platform task, an adaptation of the Barnes maze. Measures of hippocampal plasticity, however, were not investigated. Also at the behavioral level, RSV (10 and 20 mg/kg, orally administered in conjunction with 2.5 mg/kg piperine—an alkaloid that enhances the bioavailability of RSV in vivo [91]) exerted antidepressant effects in ICR mice, as measured by the reduced immobility time in both FST and TST [92]. Although AHN measures were not examined, the authors report serotonergic and noradrenergic changes in the hippocampus of RSV + piperine-treated mice, such as reduced activity of monoamine oxidase-A (MAO-A) enzyme and increases of serotonin and noradrenalin levels.

As can be noted throughout the reviewed literature, the majority of studies in healthy rodents are descriptive investigations showing possible associations between a certain RSV treatment regimen and behavioral and hippocampal changes. Not many of these associations, however, can be considered to show a causal relationship. One of the most interesting reports in the RSV literature that tried to bridge this gap is a study by Harada et al. [93]. In this study, the authors report that oral administration of RSV (20 mg/L) once daily for 3 weeks was able to induce insulin-like growth factor 1 (IGF-I) production in the hippocampus, increase AHN (as assessed by the number of BrdU^+^ cells and the number of BrdU^+^/calbindin-D28k^+^ cells) and angiogenesis (defined as the number of BrdU^+^/PECAM-1 [CD31]^+^ cells), and improve spatial learning and memory in the MWM. Interestingly, none of these effects were observed in calcitonin gene-related peptide- (CGRP-) knockout mice treated with the same regimen of RSV, whilst, in vitro, RSV increased CGRP release from dorsal root ganglion (DRG) neurons from Wt mice. Considering that RSV was undetectable in the hippocampus of RSV-treated Wt mice, the report provides strong evidence that, in vivo, RSV might exert effects on the hippocampus through stimulating the release of CGRP from DRG neurons, leading to enhanced production of IGF-I by hippocampal astrocytes, thereby improving AHN and cognitive performance.

However, not all studies report proneurogenic effects of RSV. For example, Park et al. [94] report that mice administered either 1 or 10 mg/kg RSV for two weeks had reduced numbers of both NPCs and newly generated neurons in the DG of the hippocampus relative to a vehicle-treated control group, in a dose-dependent manner. Furthermore, they also found a reduction in BDNF and pCREB in the hippocampus and impaired spatial learning in the MWM of the RSV-treated animals relative to controls. As the authors themselves observe in their discussion, there is a marked difference in the amount of RSV administered to the animals in this study (25 or 250 microg/day/mouse) compared to some of the other apparently conflicting studies such as Harada et al. [93], where a much lower dose was administered (4 microg/day/mouse). They argue therefore that there is evidence for opposite effects on hippocampal neurogenesis depending upon the dose of RSV administered. Detrimental effects of RSV to hippocampal neurogenesis also came from in vitro studies showing that RSV treatment (10, 20, and 50 *μ*M) significantly reduced the number of neurospheres derived from hippocampal precursors [95]. Furthermore, the same study showed that RSV (2 and 4 *μ*M) decreased the proportion of neurospheres expressing *β*-III tubulin, a marker of differentiated neurons.

## 4. Limitations and Perspectives

Despite the weight of evidence mostly pointing to RSV as an enhancer of hippocampal plasticity, some limitations in the recent literature can be observed. For instance, the lack of behavioral and cellular data from healthy control subjects limits our understanding of the effects of RSV in physiological conditions. In Liu et al.'s work [38], the effects of RSV in the hippocampal markers (BDNF, pERK, and pCREB) in control animals (not UCMS) are not shown. This makes it difficult to evaluate whether RSV has positive effects on the hippocampus of healthy individuals. In Xu et al.'s work [53], however, the hippocampal levels of pERK in vehicle-treated mice are augmented as a result of RSV treatment, even in the absence of the EtOH insult. In addition, especially considering that most of the opposite effects of RSV as a neural plasticity enhancer come from in vivo and in vitro studies that do not mimic any particular neuropsychiatric condition (such as depression, anxiety, AD, or stroke), further replication and novel investigations of the effects of this polyphenol on AHN and other aspects of hippocampal plasticity in the healthy brain are imperative.

Some discrepancies are also found in the context of diabetes. In this respect, some encouraging findings by Jing et al. [75] and Thomas et al. [76] point to a potential role of RSV for enhancing AHN in diabetic rodents. These findings, however, are not in full alignment with those by Venturini et al. [77], where RSV supplementation was not able to elicit changes to the lower levels of cell proliferation in the hippocampus of diabetic rats. This incongruence could be due to a number of factors, including the rodent strain (Sprague-Dawley rats versus C57BL/6 mice versus Wistar rats) and the regimen (0.75 mg/kg (oral), 3x/day for 4 weeks versus 50 mg/kg/day for 6 weeks versus 20 mg/kg for 3 weeks). Furthermore, Venturini et al. [77] could not find differences in the number of proliferating cells in the DG, but other stages of AHN—such as neuronal differentiation, maturation, and survival—were not investigated. A previous study using an animal model of generalized anxiety disorder, rather than the streptozotocin-induced diabetes model, showed differences in other stages of AHN despite similar levels of cell proliferation in the DG [96]. Therefore, the potential role of RSV as an AHN enhancer in the context of diabetes could still be valid and deserves further investigation.

One of the key aspects in any attempt to determine how RSV affects hippocampal plasticity relates to the controversial roles of this polyphenol's receptor, Sirt1. For instance, in a study by Xu et al. [53], RSV is shown to be able to antagonize the detrimental effects of EtOH on NPCs and neurogenesis, a finding accompanied by the rescue of hippocampal levels of pERK, Hes 1, and Sirt1. The authors point to the involvement of these proteins in the regulation of NPCs; however, Saharan et al. demonstrated that Sirt1 signaling negatively regulates neuronal differentiation in the adult hippocampus, with Sirt1 knockdown increasing the neurogenic potential of NPCs in vivo and in vitro [95]. In their discussion, the authors also claim that previous studies have reported that Sirt1 and Hes 1 interact and repress downstream targets [97], probably including those involved in inhibiting neuronal differentiation [95]. Similarly, Ma et al. report increased neurogenesis in the adult hippocampus with Sirt1 knockout mice [98]. However, they report that this arises primarily as a result of the loss of the repressive effects of Sirt1 on neural stem cell self-renewal, rather than on neuronal differentiation. Interestingly, Ma et al. also report that the survival of newborn neurons in the Sirt1 knockout mice is reduced, suggesting a role for this protein in neuronal viability [98]. In light of these reports additional studies with RSV are needed to further clarify the role of Sirt1 on the reported effects of RSV on hippocampal neurogenesis.

In addition, as discussed by Hurley et al. [50], most studies utilize intraperitoneal injection regimens. For translational purposes, therefore, novel assays testing effective doses of RSV administered orally, as well as investigating their correspondence to appropriate intake in humans, are needed. In this same respect, it is worth noting the need for randomized controlled trials investigating the effects of RSV supplementation with either tablets or diet on the behaviors and brain functions discussed here. Addressing this issue, a recent double-blind placebo-controlled study showed that intake of RSV capsules for 26 weeks (200 mg per day) improved memory retention (retention of words) and enhanced hippocampal functional connectivity in healthy overweight older individuals [99]. It will be interesting to see in the future if these encouraging results could also be observed in other age and body mass index populations, especially considering that another recent randomized double-blind placebo-controlled study found that, when administered in conjunction with piperine, RSV can augment cerebral blood flow in young healthy adults [100]. Nevertheless, the study failed to demonstrate that RSV could improve mood and cognition in this population. These negative results could be due to the relatively short regimen applied: only three doses of RSV (250 mg) at least a week apart. Also concerning the use of RSV by humans, a recent report showed that a single dose of this polyphenol (500 mg tablet taken orally by healthy adults) was able to promote relevant pharmacological activities, comparable to those reported by in vitro studies [101]. It was also promising that the study revealed that besides being well absorbed, RSV was also well tolerated by all participants. However, the population studied was very small (*n* = 15 [9 males and 6 females]); therefore future replications using larger samples would be highly desirable. Another useful line of investigation for the field of RSV and neural plasticity could be to examine if this polyphenol administered in conjunction with other beneficial strategies (such as CR, physical exercise, EE, and even other polyphenols) could exert synergistic effects capable of amplifying the potential enhancement of hippocampal plasticity observed in most of the RSV studies.

## 5. Conclusion

Overall, for the neuropsychiatric conditions discussed here—depression, anxiety, stroke, diabetes, EtOH administration, chronic fatigue, and AD—RSV appears to be, at least in rodents, an effective agent in promoting neuroprotection and hippocampal plasticity, including aspects of AHN. Nevertheless, the literature is not completely consistent in providing conclusive evidence pointing to RSV as a plasticity/AHN-enhancer. These opposing effects were mainly observed in assays attempting to evaluate the physiological effects of RSV (i.e., not using specific models of diseases) and deserve attention before it can be affirmed that RSV is a safe proplasticity agent. Novel studies addressing the limitations discussed in the present review are therefore needed so that a better understanding of the circumstances—dose, condition (neuropathology model versus healthy brain), form of administration, and treatment duration—in which RSV is beneficial to brain plasticity can be achieved.

## Figures and Tables

**Table 1:** Bibliographical search results conducted in PubMed/Medline and Web of Science databases, using the keywords “resveratrol AND hippocampal neurogenesis” and “resveratrol AND hippocampal plasticity” (May 27, 2015).

Databases	Resveratrol AND hippocampal neurogenesis	Resveratrol AND hippocampal plasticity
PubMed/Medline	12	6
Web of Science	12	9

**Table 2:** Effects of resveratrol on hippocampal plasticity.

Model	Condition studied	RSV treatment	Effects of RSV on behavior	Cellular and molecular effects of RSV in the hippocampus	Conclusion/proposed mechanism	Reference
Male Wistar rats (180–200 g)	Depression (UCMS)	20, 40, and 80 mg/kg (daily i.p. injections, for 5 weeks)	↑ sucrose consumption (all doses); ↓ immobility time in the FST (40 and 80 mg/kg); ↑ locomotor activity and grooming in the OFT (80 mg/kg)	↑ BDNF (80 mg/kg), pERK (80 mg/kg), and pCREB (40 and 80 mg/kg) levels to control levels	Antidepressant effects of RSV likely to be mediated by its ability to regulate HPA axis function and ↑ BDNF, pERK, and pCREB levels in the hippocampus and amygdala	[38]

Male Wistar rats (180–200 g)	Depression (UCMS)	80 mg/kg (daily i.p. injections, for 5 weeks)	RSV prevented stress-induced impairment of spatial working memory in the MWM and recognition memory performance in the NORT	↑ BDNF, pERK, and pCREB levels to control levels	RSV can prevent UCMS-induced cognitive impairment likely via modulating HPA axis function and ↑ BDNF, pERK, and pCREB levels in the hippocampus and PFC	[40]

Male Wistar rats (250–300 g)	Depression (UCMS)	5 mg/kg or 20 mg/kg (daily i.p. injections, for 35 days)	RSV did not prevent the UCMS-induced decrease in locomotor activity; RSV (20 mg/kg) prevented memory impairment in the PAT and MWM	↑ BDNF and c-Fos levels in CA1 and CA3 to nonstressed control levels	RSV can prevent UCMS-induced cognitive impairment likely via ↑ expression of BDNF and c-Fos in the hippocampus and regulating plasma levels of TNF-*α* and IL-*β*	[41]

4–6-week-old male Swiss albino mice	Depression (s.c. injection of CORT [40 mg/kg] for 21 days)	80 mg/kg (oral), 30 min prior to CORT injection, for 21 days	↑ sucrose consumption; ↓ immobility time in the FST and TST	↑ BDNF levels	RSV exerts antidepressant effects, likely through restoration of the HPA axis and upregulation of hippocampal BDNF	[47]

Offspring (PND 40) of 3-4-month-old Wistar rats	Prenatal stress (restraint stress during early or late gestational period)	10 mg/kg (oral) throughout pregnancy	N/A	↑ number of DCX^+^ neurons and ↑ expression of BDNF	Effects of RSV likely to occur via SIRT-1-induced activation of AMPK, which stimulates neuronal differentiation and mitochondrial biogenesis. This, in turn, could lead to ↑ BDNF activation via ↑ production of ATP	[49]

14-15-week-old Wistar-Kyoto rats	Noninduced model of depression	10 or 40 mg/kg (acute or chronic—daily i.p. injections, for 7 days)	↓ immobility time in the FST (acute, 40 mg/kg, and chronic, 10 and 40 mg/kg); no effects in the OFT; ↑ sucrose intake (chronic, 10 and 40 mg/kg)	↑ BDNF expression (chronic, 10 and 40 mg/kg)	Effects of RSV likely to occur via activation of BDNF	[50]

In vivo: PND6-PND14 C57/BL6 mice In vitro: C17.2 cells	EtOH exposure	In vivo: 20 mg/kg i.p. injection at PND6 (followed by two 2.5 g/kg i.p. injections of 20% EtOH at PND7) In vitro: 5 *µ*M (for 12 h); EtOH (700 mg/dL) added after 12 h RSV treatment (and kept for 12 h or 36 h)	N/A	In vivo: RSV rescued ↓ in the number of (DG): granule cells; BrdU^+^, Sox2^+^, Sox2 & GFAP^+^, BLBP & nestin^+^, BrdU & DCX^+^, BLBP & GFAP^+^ cells; spine density; mushroom spine proportion; (hippocampus) pERK/ERK ratio; Hes 1 and Sirt1 protein levels In vitro: ↑ ratio of Ki-67^+^ cells; reversed cell apoptosis and cell arrest of NPCs	EtOH-mediated ↓ in postnatal hippocampal neurogenesis likely involves expression of pERK and Hes 1 in the neonatal hippocampus; activation of SIRT1 by RSV can protect neonatal neurogenesis from EtOH-induced detrimental effects	[53]

8-week-old female BALB/c mice	CFS	40 mg/kg (oral), daily for 4 weeks	↑ daily activity	RSV rescued ↓ in the number of BrdU^+^ cells; ↓ apoptosis in the DG; ↑ BDNF mRNA expression; ↓ levels of acetylated p53	RSV can potentially improve fatigue symptoms and enlarge the CFS-related atrophic hippocampus likely through ↓ apoptosis and ↑ cell proliferation in the DG	[60]

Male Wistar rats (325–375 g)	GCI	1 or 10 mg/kg daily i.p. injections for 21 days prior to surgery	Both doses of RSV ↑ swimming time in the target quadrant during the probe trial of the MWM 7-8 weeks following GCI	Both doses of RSV led to ↑ CA1 neuronal density (7 and 85 days after GCI); ↓ DCX/PSA-NCAM colabeled cells in the DG (both doses and at both intervals); ↑ expression of CD31 in CA1 (1 mg/kg at 7 days); ↑ expression of CD31 in CA1 (1 and 10 mg/kg at 85 days); ↑ expression of CD31 in CA3 (1 and 10 mg/kg at 7 days); ↓ expression of CD31 in the GD (1 mg/kg at 7 days); ↑ expression of CD31 in the DG (1 and 10 mg/kg at 85 days)	Chronic RSV administration is associated with neuroprotection against GCI likely through restoration of AHN levels and increased angiogenesis	[65]

Male SD rats (250–280 g)	Poststroke depression	10, 20, or 40 mg/kg (gavage), once daily 7 days prior to MCAO and 1 day or 13 days after insult	RSV (20 and 40 mg/kg) ↑ sucrose preference 13 days after MCAO and ↓ immobility time in the FST	RSV (20 and 40 mg/kg) ↓ CRF protein expression, restored expression of GR, and ↑ BDNF protein expression	RSV exerts neuroprotective effects against stroke and poststroke depression in part mediated by HPA axis regulation	[69]

8–10-week-old male SD rats	Diabetes (streptozotocin-induced)	0.75 mg/kg (oral), 3x/day (8 h interval) for 4 weeks	N/A	↓ number of degenerative neurons in CA3; ↓ astrocytic activation in CA1 and CA3; ↓ hippocampal expression of TNF-*α*, IL-6, pERK1/2, and phospho-p38; ↓ BBB permeability and VEGF, both in the hippocampus; ↑ mitochondrial genesis in CA3 neurons; ↑ hippocampal pAMPK	RSV could be effective for treating diabetes due to its anti-inflammatory/antineurodegeneration effects in the hippocampus	[75]

7-week-old C57BL/6 mice	Diabetes (streptozotocin-induced)	50 mg/kg mixed with AIN93G diet per day for 6 weeks	N/A	↑ expression of Hdac4 and Jak1 genes; ↓ expression of ApoE and Hat1 genes, in comparison with non-RSV diabetic mice	RSV could be effective for cognitive function in diabetes due to its effects in normalizing the expression of AHN and synaptic plasticity genes in the hippocampus	[76]

Male Wistar rats (250–300 g)	Diabetes (streptozotocin-induced)	20 mg/kg (gavage) for 3 weeks	N/A	RSV could not restore the lower levels of hippocampal cell proliferation (number of BrdU^+^ cells)	RSV was effective in promoting antioxidant effects in diabetic rats but failed to enhance AHN	[77]

In vitro: (E7) rat hippocampal H19-7 neuronal cell line	AD	75 *µ*M for 2 h before 25 *µ*M of A*β* for 24 h	N/A	RSV ↑ expression of PSD-95, Arc, and synaptophysin	RSV's neuroprotective effects over memory loss in vitro might occur through improvement of expression of memory-associated proteins	[82]

21-month-old male Fischer 344 rats	Aging	40 mg/kg (daily i.p. injections) for 4 weeks (analysis at 25 months of age)	↑ learning and memory in the MWM; ↓ immobility time in the FST	↑ AHN (↑ number of BrdU^+^ cells; net BrdU^+^/NeuN^+^; ↑ number of DCX^+^ cells); ↑ RECA-1 in CA1 and entire hippocampus; ↓ hypertrophy of astrocytes; ↓ microglia activation	RSV administered in late middle age might ↑ memory and mood likely through modulation of synaptic plasticity and suppression of inflammation	[83]

20–22 g ICR mice	—	1.25, 2.5, 5, 10, and 20 mg/kg + piperine (2.5 mg/kg)	↓ immobility time in the FST and TST (10 and 20 mg/kg RSV + 2.5 mg/kg piperine)	↓ MAO-A activity; ↑ 5-HT and NE	Antidepressant effects of RSV combined with piperine may be due to activation of the 5-HT and NE systems in brain regions including the hippocampus	[92]

4-week-old male C57BL/6 mice	—	1 or 10 mg/kg i.p. injection/day for 14 days	↑ latency to find the hidden platform of the MWM	↓ number of DCX^+^ cells and of BrdU^+^/NeuN^+^ in the DG; ↓ BDNF and pCREB levels	RSV impairs AHN, likely through suppression of CREB and BDNF	[94]

In vitro: 14-day hippocampal neurospheres from adult male C57Bl/6 mice	—	0.1, 1, 3, 5, 10, 20, and 50 *µ*M (assessment of number of neurospheres/hippocampus) 0.5, 1, 2, and 4 *µ*M (assessment of proportion of neurospheres expressing *β*-III tubulin)	N/A	↓ number of neurospheres/hippocampus (10, 20, and 50 *µ*M RSV); ↓ proportion of neurospheres expressing *β*-III tubulin (2 and 4 *µ*M)	RSV hinders differentiation of neurons from adult neural precursors, likely through activation of Sirt1 signaling	[95]

AD = Alzheimer's disease; AMPK = 5′ adenosine monophosphate-activated protein kinase; APOE = apolipoprotein E; Arc = activity-regulated cytoskeleton-associated protein; ATP = adenosine triphosphate; BBB = blood-brain barrier; BDNF = brain-derived neurotrophic factor; BLBP = brain lipid-binding protein; BrdU = bromodeoxyuridine; CFS = chronic fatigue syndrome; CORT = corticosterone; CRF = corticotropin-releasing factor; DCX = doublecortin; DG = dentate gyrus; E7 = embryonic day 7; EtOH = ethanol; 5-HT = 5-hydroxytryptamine; FST = forced swimming test; GCI = global cerebral ischemia; GFAP = glial fibrillary acidic protein; GR = glucocorticoid receptor; HAT1 = histone acetyltransferase 1; HDAC4 = histone deacetylase 4; HPA = hypothalamic-pituitary-adrenal; IL-1*β* = interleukin-1*β*; IL-6 = interleukin-6; JAK1 = Janus kinase 1; MAO-A = monoamine oxidase A; MCAO = middle cerebral artery occlusion; N/A = not assessed; NE = noradrenaline; NeuN = neuronal nuclei protein; NORT = novel object recognition task; OFT = open field test; PAT = passive-avoidance test; PFC = prefrontal cortex; PND = postnatal day; PSD-95 = postsynaptic density protein 95; RECA-1 = endothelial cell antigen-1; RSV = resveratrol; S.C. = subcutaneous; SD = Sprague-Dawley; SIRT1 = nicotinamide adenine dinucleotide-dependent deacetylase sirtuin-1; TNF-*α* = tumor necrosis factor-*α*; TST = tail suspension test; UCMS = unpredictable chronic mild stress; VEGF = vascular endothelial growth factor.
